# Discordance between Adolescents and Parents in Functional Somatic Symptom Reports: Sex Differences and Future Symptom Prevalence

**DOI:** 10.1007/s10964-023-01810-w

**Published:** 2023-06-24

**Authors:** Elske Hogendoorn, Aranka V. Ballering, Marijn W. G. van Dijk, Judith G. M. Rosmalen, Sarah M. Burke

**Affiliations:** 1 University of Groningen, University Medical Center Groningen, Department of Psychiatry, Groningen, The Netherlands; 2 University of Groningen, Department of Developmental Psychology, Heymans Institute for Psychological Research, Groningen, The Netherlands; 3 University of Groningen, University Medical Center Groningen, Department of Internal Medicine, Groningen, The Netherlands

**Keywords:** Functional somatic symptoms, Sex differences, Gender roles, Adolescence, Parents, Informant discordance

## Abstract

Functional somatic symptoms, i.e., physical complaints that cannot be sufficiently explained by an objectifiable biomedical abnormality, become increasingly more prevalent in girls than in boys during adolescence. Both parents and adolescents report more functional somatic symptoms in girls, but their reports correspond only limitedly. It remains unknown whether parent-adolescent discordance contributes to the higher symptom prevalence in girls. This study investigated parent-adolescent discordance in reported functional somatic symptoms throughout adolescence, examined the longitudinal association of parent-adolescent discordance with symptom prevalence in early adulthood and focused on sex differences in these processes. Participants included 2229 adolescents (50.7% female) from four assessments (age 11 to 22 years) of the TRAILS population cohort. Parents and adolescents reported significantly more symptoms in girls than in boys during adolescence. Variance analyses showed that throughout adolescence, parents reported fewer symptoms than girls self-reported and more than boys self-reported. Regression analyses using standardized difference scores showed that lower parent-report than self-report was positively associated with symptom prevalence in early adulthood. Polynomial regression analyses revealed no significant interaction between parent-reported and adolescent self-reported symptoms. Associations did not differ between boys and girls. The findings show that lower parent-reported than self-reported symptoms predict future symptom prevalence in both sexes, but this discordance was more observed in girls. The higher functional somatic symptom prevalence in girls might be partly explained by parental underestimation of symptoms.

## Introduction

During adolescence, there is a growing difference between boys’ and girls’ reporting of functional somatic symptoms (i.e., physical complaints that cannot be sufficiently explained by a detectable biomedical abnormality), with girls reporting more symptoms than boys (Janssens et al., 2011). Gendered parenting with regard to symptoms in adolescence may be a factor contributing to this sex difference. Gaining more insight into discordance in parent-reported and adolescent boys’ and girls’ self-reported functional somatic symptoms is crucial to understand the higher symptom prevalence in girls and targeting parents for interventions. However, the majority of studies only include either parent-report or self-report rather than a combination, and lack follow-ups into adulthood. Thus, the current four-wave longitudinal study aimed to investigate parent-adolescent discordance in reporting of functional somatic symptoms in boys and girls over the course of adolescence and its association with symptom prevalence in early adulthood.

Functional somatic symptoms are defined as physical symptoms, for example headache, back pain or tiredness, that cannot be entirely attributed to a detectable biomedical abnormality after adequate diagnostic research and history taking (Beck, 2008). Experiencing functional somatic symptoms from time to time is normal and these complaints usually spontaneously disappear. However, 4% of adolescents experience persistent functional somatic symptoms (Janssens et al., 2014). Persistent symptoms in adolescence are associated with physical impairment, deteriorated school functioning, and social withdrawal (Youssef et al., 2006). Girls tend to consistently report more functional somatic symptoms than boys (Romero-Acosta et al., 2013; van Geelen et al., 2015). The sex difference in symptom prevalence is already present in childhood and tends to increase during adolescence (LeResche et al., 2005). This could be due to biological dissimilarities, as during the adolescent period many physical changes take place, which possibly influence somatic symptom proneness (Vogt Yuan, 2007).

Aside from differences in biological vulnerability for somatic symptoms, the sex difference in symptom prevalence may relate to psychosocial differences. Psychosocial differences between boys and girls can be described in terms of gender. Gender is an umbrella term entailing the embodiment of different identities, roles, behaviors and relationships of men and women prescribed by societal norms in a given time and society, whereas sex refers to biological characteristics, including hormones and anatomy of male and female bodies (Smith & Koehoorn, 2016). Research has shown that in adults, gender associates with somatic symptoms independently of sex, which is possibly due to the adherence to normative gender roles (Ballering et al., 2020). The traditional masculine gender roles includes stoicism, high pain tolerance and not showing weakness, whereas feminine gender roles allow for vulnerability and expression of pain (Pool et al., 2007). During adolescence, most boys and girls behave increasingly according to their socially-prescribed gender roles (Chaplin & Aldao, 2013). In addition, adolescents generally become more independent of their parents or caregivers (henceforth referred to as parents) and spend more time with predominantly same-sex peers, which encourages adherence to gender roles (Lam et al., 2014; Witt, 2006). A qualitative study revealed that adolescents are aware of gender role expectations regarding somatic symptoms and that they feel pressured to adhere to these, especially when among peers (MacLean et al., 2010). Thus, these processes of gendered socialization in adolescence may be related to symptom reporting in boys and girls.

Gender role patterns regarding symptoms may be transmitted from parents to their children through family upbringing. Social learning of illness behavior begins in childhood. From an early age onwards, children learn to interpret physical sensations, give meaning to them and respond to them by observing and communicating with their caregivers (Levy et al., 2000). As such, parents’ management of their child’s symptoms may influence the child’s interpretation, communication and management of future symptoms. Consistent with gender role expectations, parents encourage more independence and control of emotions (“being tough”) in sons regarding symptoms, while they behave more protectively towards daughters, and encourage daughters to share their feelings and symptom experiences (Clementi et al., 2019; Walker & Zeman, 1992). Any reaction to a symptom is preceded by an assessment of that symptom. The way parents assess their child’s symptoms differs for boys and girls, with meta-analytic evidence showing that parents report more functional somatic symptoms in girls than in boys, possibly reflecting parental beliefs about gender roles (Duhig et al., 2000).

However, the previous meta-analysis and subsequent studies did not include adolescents’ self-reported functional somatic symptoms (Duhig et al., 2000; Janssens et al., 2014). Yet, prior studies have found that parent-reports and child self-reports on somatic symptoms correspond only to a limited extend (De Los Reyes et al., 2015; Hart et al., 2013). In addition, it has been shown that parent-child discordance in psychopathology predicts several clinical features, such as emotional and behavioral problems and social competence (Guion et al., 2009; Van De Looij-Jansen et al., 2011). Combining both parent and child perspectives is clinically relevant and useful to inform preventive, diagnostic and treatment strategies for functional somatic symptoms (Kraemer et al., 2003). Previous studies have identified high parent-reported functional somatic symptoms as a risk factor for persistence of symptoms, but disregarded sex differences and included follow-ups to mid-adolescence instead of adulthood (Janssens et al., 2014). Parent-child discordance in functional somatic symptoms may reflect gendered parenting. This is indicated by the finding that parents behave differently towards sons and daughters when they experience somatic symptoms, and may thus perceive symptoms differently (Clementi et al., 2019). It has also been suggested that parents are more likely to report symptoms in their children that are congruent with gender expectations (Schroeder et al., 2010). Parent-adolescent discordance in symptom reporting could thus be informative in studying sex differences in functional somatic symptoms. It would be highly valuable to gain more insight into the course of (discordance between) parent- and self-reported functional somatic symptoms throughout adolescence, its association with symptom prevalence in adulthood, and sex differences herein.

## The Current Study

There is a paucity of studies that combine parent and adolescent perspectives and examine sex differences in the longitudinal course and associations of parent-adolescent discordance in symptom reporting. Therefore, it remains unclear whether parent-adolescent discordance contributes to the higher functional somatic symptom prevalence in girls. This study sought to address the gaps in the existing literature by taking into account the sex of the adolescent, including both parent-reported and adolescent self-reported functional somatic symptoms and discordance herein, and by adopting a longitudinal approach with follow-ups into early adulthood. This study examined differences in functional somatic symptoms in boys and girls over the course of adolescence, studying parent-report and self-report (aim 1). Furthermore, this study investigated if parent-adolescent discordance changes over time in adolescence and differs between boys and girls (aim 2). Lastly, longitudinal associations were investigated between parent-adolescent discordance and symptom prevalence in early adulthood, and sex differences herein (aim 3). This was studied in a large population-based cohort using four assessment waves in adolescence and early adulthood. Based on literature on gendered parenting, it was hypothesized that parent-adolescent discordance in reported functional somatic symptoms is larger in adolescent girls than in boys, with parents perceiving more symptoms in their daughters than their sons (hypothesis 1). Furthermore, this study hypothesized that the course of parent-adolescent discordance is different for boys and girls (hypothesis 2). Lastly, based on literature indicating that parent-child discordance in psychopathology contributes to future poor outcomes, it was expected that parent-adolescent discordance predicts symptom prevalence in early adulthood (hypothesis 3a), and that increased parental reporting of functional somatic symptoms in girls, compared to adolescent self-report, contributes to the higher symptom prevalence in girls in early adulthood (hypothesis 3b).

## Method

### Sample and Procedure

This study is part of the Tracking Adolescents’ Individual Lives (TRAILS) study. TRAILS is an ongoing prospective cohort study that investigates mental health and social development from pre-adolescence onwards. TRAILS-participants lived in one of the three northern provinces of the Netherlands at the time of recruitment and were intended to represent the general Dutch preadolescent population. Participants were recruited through primary schools. Primary schools that participated in TRAILS were comparable to other primary schools in the Netherlands with regard to the proportion of children with a low socioeconomic background. Detailed information about recruitment and sample characteristics has been reported elsewhere (Oldehinkel et al., 2015). Topics of previous TRAILS studies include the use of reports of multiple informants, and have pointed out the informative nature of discrepancies in these reports, highlighting the importance of studying longitudinal associations of discordance among informants (Noordhof et al., 2008).

Participants enrolled in the TRAILS study at age 10–12 years. Measurement waves have been taking place bi- or triennially. In the current study, data from T1 (mean age 11.1 years, 51% female), T2 (mean age 13.6 years, 51% female), T3 (mean age 16.3 years, 52% female) and T5 (mean age 22.3 years, 53% female) were used from the complete sample (*n* = 2230, of which one parent-child dyad had their data deleted upon parental request, resulting in a sample size of 2229).

The Dutch Central Committee on Research Involving Human Subjects (CCMO) granted ethical approval for the TRAILS study (#NL38237.042.11). Parents provided written informed consent at T1. At T2, T3 and T5, written informed consent was also obtained from the TRAILS-participant.

### Measures

Main variables of the study involved self-reported and parent-reported functional somatic symptoms. SES, pubertal status and gender non-contentedness were included as covariates as previous studies indicated these may be relevant to sex differences in parent-adolescent discordance of somatic symptoms (Brix et al., 2019; Hinz et al., 2017; Janssens et al., 2011; Roubinov & Boyce, 2017).

#### Self-reported functional somatic symptoms

Self-reported functional somatic symptoms were measured using the Somatic Complaints subscale of the Youth Self Report (YSR) (Achenbach & Rescorla, 2001) at T1, T2 and T3. At T5, the Adult Self Report (ASR) (Achenbach & Rescorla, 2003) was used, which was appropriate for the age of the participants at that time. This subscale contains items that refer to somatic complaints without a known medical cause or without obvious reason. The TRAILS-participant indicated to what extent each complaint had applied to him/her in the prior six months. Answers were rated on a three-point Likert scale (0 = not at all true; 1 = sometimes true; 2 = often true). Two items, ‘eye problems’ and ‘skin problems’, were excluded since previous TRAILS studies reported low factor loadings, indicating that these two items did not represent the underlying construct of functional somatic symptoms well in the TRAILS cohort (Janssens et al., 2010, 2014). Moreover, three items of the ASR (heart pounding, numbness, and trouble sleeping) were excluded to ensure consistency with the YSR. The remaining seven items included dizziness, overtiredness, aches/pains, headache, nausea, stomach pain, and vomiting. Both the YSR and ASR show adequate reliability, validity and measurement invariance (Achenbach et al., 2016; Fonseca-Pedrero et al., 2012; Najman et al., 2008). Internal consistency (measured by Cronbach’s alpha) of the seven items used in our study was good (T1: α = 0.76; T2: α = 0.77; T3: α = 0.75; T5: α = 0.71).

#### Parent-reported functional somatic symptoms

Parent-reported functional somatic symptoms were assessed at T1, T2 and T3 using the Somatic Complaints subscale of the Child Behavior Checklist (CBCL) (Achenbach, 1991). The CBCL corresponds to the YSR and ASR presented in the form of parent-report. The parent of the TRAILS-participant stated to what extent each complaint had applied to their child in the prior six months on a three-point Likert scale with the same scoring categories. The item ‘obstipation’ was excluded from the parent-reported data since it is not part of the YSR/ASR, leaving only the corresponding items. Psychometric properties are good and the CBCL has been validated in numerous populations (Fombonne, 1991; Verhulst et al., 1985). Internal consistency of the seven items used in our study was good (T1: α = 0.71; T2: α = 0.72; T3: α = 0.73).

#### Sex

Sex of the TRAILS-participant was dichotomously assessed at T1 using self-report (male/female).

#### Gender non-contentedness

In this study, gender non-contentedness refers to any expressed or felt desire to be of the opposite gender or sex. Gender non-contentedness was measured at T1, T2, T3 and T5 with the item ‘I wish to be of the opposite sex’ of the YSR or ASR. Adolescents experiencing gender non-contentedness might show sex-incongruent gender role behaviors with regard to their symptoms, which also might influence parents’ assessment of their child’s symptoms. The TRAILS-participant indicated to what extent this statement had applied to him/her in the prior six months on a 3-point Likert scale (0 = not at all true; 1 = sometimes true; 2 = often true). Gender non-contentedness was defined as a score equaling or exceeding 1.

#### Socioeconomic status

Socioeconomic status (SES) was assessed at T1 by calculating the average of the z-scores of the following indicators: educational and occupational level of each parent, and household income. Z-scores were calculated based on the International Standard Classification of Occupations (Ganzeboom & Treiman, 1996).

#### Pubertal status

Pubertal status was measured at T1, T2 and T3 using the Tanner Scale of Pubertal Status (Marshall & Tanner, 1969, 1970). The Tanner Scale includes five stages, with a higher stage referring to a later stage of development. At T1, the parent of the TRAILS-participant was surveyed about two physical characteristics of their child. Using schematic drawings, they reported on genital development and pubic hair for boys, and breast development and pubic hair for girls. For each characteristic, the parent rated which of the five Tanner stages was most applicable to the TRAILS-participant. At T2 and T3, pubertal status was assessed by a self-reported five-item questionnaire. The characteristics for boys comprised growth spurt, skin changes, body hair, voice-change, and facial-hair growth. The characteristics for girls included growth spurt, skin changes, body hair, breast development, and menarche. Answers were rated on a 4-point Likert-scale (0 = not yet started; 1 = barely started; 2 = definitely started; 3 = seems complete), except for menarche, which was assessed dichotomously (0 = no, 1 = yes).

### Statistical Analyses

This study was preregistered prior to analysis of the data (https://osf.io/cbrqa). Characteristics of the study sample are presented per assessment wave. According to the SAGER guidelines, results were stratified by sex if applicable (Heidari et al., 2016).

First, to examine if parents and adolescents perceive functional somatic symptoms differently in boys than in girls over the course of adolescence (aim 1), correlations between parent-reported and self-reported functional somatic symptoms at T1, T2 and T3 were calculated for boys and girls separately. Independent T-tests were performed to assess whether parent-reported and self-reported functional somatic symptoms at T1, T2 and T3 differed statistically significantly between boys and girls. Subsequently, standardized difference scores were calculated by subtracting standardized parent-reported symptom scores from standardized self-reported symptom scores at T1, T2 and T3. Then, to assess whether the standardized difference between parent-reported and adolescent-reported symptoms differed per sex of the adolescent, ANCOVA tests were performed at T1, T2 and T3 with sex as fixed effect and parent-adolescent discordance as dependent variable. SES, pubertal status and gender non-contentedness were included as covariates.

Second, sex differences in changes in parent-adolescent discordance over the course of adolescence were tested (aim 2) using a mixed model ANOVA. Sex was included as between-subjects factor, time as within-subjects factor, and parent-adolescent discordance as dependent variable. The repeated measures of T1, T2 and T3 were used.

Lastly, it was examined whether differences in parent-reports and adolescent self-reports of functional somatic symptoms provide information in the prediction of symptom prevalence in early adulthood (aim 3). Two statistical approaches were applied to incorporate informant discordance in the prediction of later symptom prevalence. According to an earlier study, using standardized difference scores when predicting health outcomes requires caution, because it may yield inaccurate results due to unequal variability and different bivariate associations in reports of different informants (Laird & De Los Reyes, 2013). Examination of informant interaction terms in a polynomial regression framework is therefore recommended. Yet, another study compared the use of standardized difference scores and polynomial regression, and concluded that both approaches can be used complementary to each other to provide more nuanced and comprehensive results regarding discordance in informant reporting (Tackett et al., 2013). An advantage of combining both approaches is that two slightly distinct hypotheses are tested, i.e., whether a mere difference between informant reports associates with the outcome (using standardized difference scores), and whether the association between the report of one informant and the outcome varies as a function of the report of the other informant (using polynomial regression). Therefore, in the current study, it was examined if parent-adolescent discordance at T1 through T3 predicted functional somatic symptom prevalence at T5 using both approaches.

In the first part, using the method of standardized difference scores, it was initially planned to regress symptom prevalence at T5 on parent-adolescent discordance at T1–T3 in a multi-level model, to account for dependency of residual errors. The model did, however, not converge when doing so. Therefore, deviating from the preregistration, the same regression was conducted but now in a linear regression model. First, the assumption of independent residuals of the repeated measures of functional somatic symptoms was checked using visual inspection of scatterplots and the Durbin-Watson statistic. Parent-adolescent discordance at T1–T3, expressed in the standardized difference score of parent-reported and self-reported functional somatic symptoms at T1–T3, was included as independent variable and self-reported functional somatic symptoms at T5 as dependent variable. Adolescent sex, SES, pubertal status and gender non-contentedness were included as covariates. Parent-adolescent discordance at T1–T3 by adolescent sex was entered as interaction term, to test whether the association differed for boys and girls. All continuous predictors were standardized before including them in the model.

In the second part, polynomial regression analyses were conducted, again using symptom prevalence at T5 as dependent variable and adolescent sex, SES, pubertal status and gender non-contentedness as covariates. Now, parent-reported functional somatic symptoms at T1–T3 and self-reported functional somatic symptoms at T1–T3 were included as separate independent variables (instead of a combination resulting in one variable reflecting parent-adolescent discordance). First, the assumption of independent residuals was checked. Second, the effects of covariates were entered (block 1), followed by the main effects 1, the quadratic main effects 2 and linear interaction terms 3 (block 2). Third, cubic main effects 4 and quadratic interaction terms 5 were entered (block 3), but dropped again if model fit did not significantly improve and none of the additional interaction terms was significant. In post-hoc polynomial regression analyses, it was assessed if the longitudinal associations between parent-reported functional somatic symptoms at T1–T3 and self-reported functional somatic symptoms at T5 differed for boys and girls, by adding an interaction between parent-reported functional somatic symptoms at T1–T3 and sex. Corresponding higher-order terms were entered in the same block-wise manner as in the main analyses.

Analyses were conducted in SPSS version 28 (IBM Corp, 2019). Unstandardized regression coefficients with 95% confidence intervals and *p*-values are reported. An α-level of 0.005 was applied to correct for multiple testing.

### Multiple Imputation of Missing Data

The percentages of missing data for self-reported functional somatic symptoms were 5.2% at T1, 6.2% at T2, 12.3% at T3, and 16.2% at T5. The percentages of missing data for parent-reported functional somatic symptoms were 11.3% at T1, 14.3% at T2, and 19.5% at T3. Missingness is unlikely to be completely random. Therefore, multiple imputation was applied in the longitudinal analyses to minimalize the risk of bias. Five data sets were generated using the Series Mean Imputation procedure in SPSS. All data sets were analyzed in an identical way, whereafter the results were pooled using Rubin’s rules (Rubin, 1987).

## Results

### Descriptive Results

Table 1 shows the characteristics of the study sample stratified by assessment wave and sex. Both parent-reported and self-reported functional somatic symptoms were significantly higher in girls than in boys at all three waves (T1–T3). As shown by sex-stratified Pearson correlations, parent-reported and self-reported functional somatic symptoms were significantly positively correlated at weak to moderate strength at all three waves (see Appendix Table 4).

**Table 1:** Descriptives of the study sample

		T1	T2	T3	T5
Boys	*n* (%)^a^	1098 (49.26)	1054 (49.07)	867 (47.69)	843 (47.33)
	Age – M (SD)	11.13 (0.56)	13.57 (0.52)	16.28 (0.71)	22.34 (0.63)
	SES – M (SD)	−0.07 (0.82)	n/a	n/a	n/a
	Self-reported FSS – M (SD)^b^	3.05 (2.41)^d^	2.17 (2.26)^e^	1.59 (1.85)^f^	1.15 (1.50)
	Parent-reported FSS – M (SD)^c^	1.29 (1.73)^g^	1.08 (1.57)^h^	0.78 (1.29)^i^	n/a
	Pubertal status – M (SD)	1.71 (0.58)	2.61 (1.08)	2.72 (0.79)	n/a
	Gender non-contentedness – *n* (%)^a^	125 (11.8)	45 (4.4)	28 (3.6)	12 (1.8)
Girls	*n* (%)^a^	1131 (50.74)	1094 (50.93)	951 (52.31)	938 (52.67)
	Age – M (SD)	11.09 (0.55)	13.57 (0.54)	16.28 (0.71)	22.25 (0.67)
	SES – M (SD)	−0.03 (0.78)	n/a	n/a	n/a
	Self-reported FSS – M (SD)^b^	3.45 (2.47)^d^	3.22 (2.55)^e^	3.09 (2.51)^f^	2.61 (2.34)
	Parent-reported FSS – M (SD)^c^	1.54 (1.81)^g^	1.52 (1.83)^h^	1.62 (2.02)^i^	n/a
	Pubertal status – M (SD)	2.01 (0.87)	3.74 (0.93)	2.84 (0.53)	n/a
	Gender non-contentedness – *n* (%)^a^	143 (12.9)	88 (8.2)	55 (6.2)	31 (3.7)

*FSS* functional somatic symptoms, *n/a* not applicable, *M* mean, *SD* standard deviation

^a^Percentage based on total sample without missing data

^b^Sum score of seven included items of the Somatic Complaints subscale of the YSR (T1–T3) or ASR (T5)

^c^ Sum score of seven included items of the Somatic Complaints subscale of the CBCL

^d^Independent T-test: *t*(2112) = 3.814, *p* < 0.001

^e^Independent T-test: *t*(2012) = 9.785, *p* <0.001

^f^Independent T-test: *t*(1592) = 13.520, *p* <0.001

^g^Independent T-test: *t*(1976) = 3.093, *p* = 0.002

^h^Independent T-test: *t*(1839) = 35.503, *p* < 0.001

^i^Independent T-test: *t*(1462) = 9.308, *p* < 0.001

### Discordance in parent- and self-reported functional somatic symptoms in adolescence

**Fig. 1:**
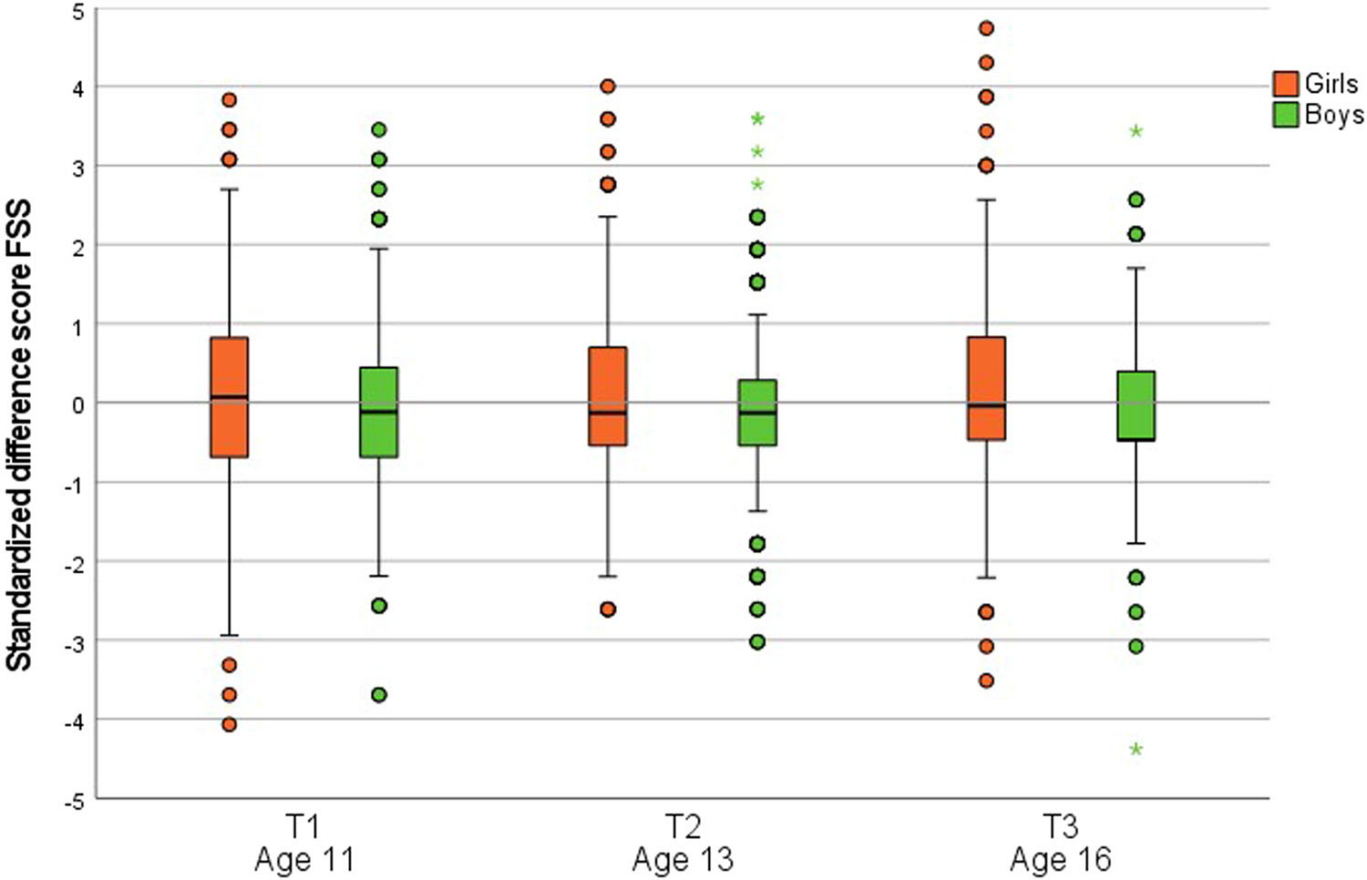
Boxplots of parent-adolescent discordance per measurement wave expressed in standardized difference scores, clustered by sex. Note. Standardized difference scores were calculated by subtracting standardized parent-reported functional somatic symptoms from standardized self-reported functional somatic symptoms

Figure 1 shows the variability of standardized parent-child discordance for boys and girls at each wave in adolescence. For both boys and girls, the medians at all waves are close to zero. Variability appears to be greater for girls.

ANCOVAs were performed per assessment wave to test if parent-adolescent discordance in functional somatic symptoms differed per sex of the adolescent. At T1, no significant association was found between sex and standardized parent-adolescent discordance after adjusting for the effect of SES, gender non-contentedness and pubertal status: *F*(1, 1815) = 0.413, *p* = 0.520. At T2 and T3, standardized parent-adolescent discordance in functional somatic symptoms differed significantly between boys and girls after adjusting for the effect of SES, gender non-contentedness and pubertal status (T2: *F*(1, 1707) = 8.312, *p* = 0.004; T3: *F*(1, 1292) = 31.359, *p* < 0.001). The standardized difference was negative in boys, indicating that parents reported more symptoms than boys themselves. In girls, the standardized difference was positive, indicating that parents reported fewer symptoms than girls themselves.

**Fig. 2:**
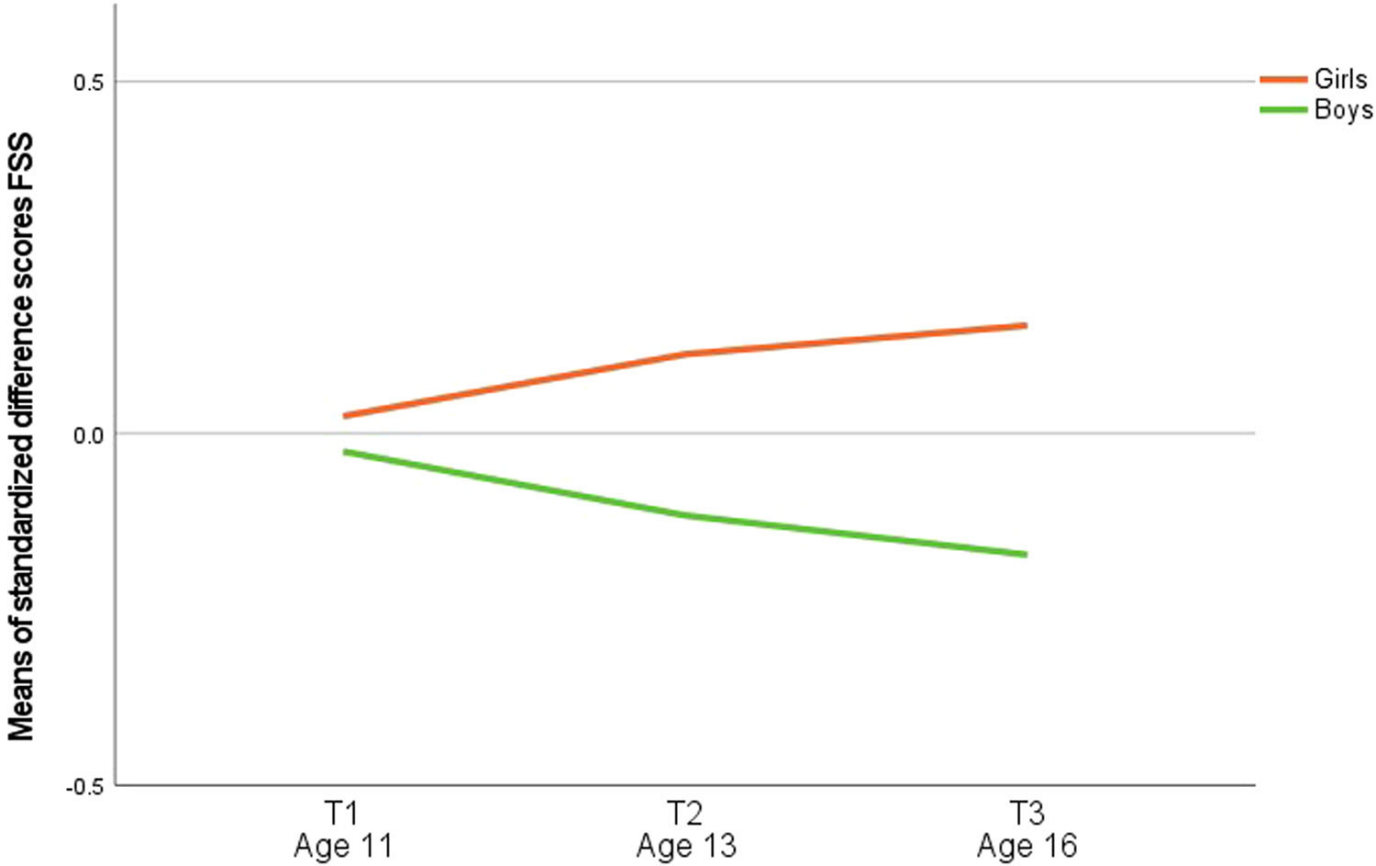
Means of the standardized difference between parent-reported and self-reported functional somatic symptoms over time, clustered by sex. Note. Standardized difference scores were calculated by subtracting standardized parent-reported functional somatic symptoms from standardized self-reported functional somatic symptoms

A mixed ANOVA was performed to test if parent-adolescent discordance changed over the course of adolescence and if this differed per sex. No significant main effect of time on parent-adolescent discordance was found (*F*(2, 2154) = 0.925, *p* = 0.397). However, both a significant main effect of sex on parent-adolescent discordance (*F*(1, 1077) = 25.779, *p* < 0.001) and a time-by-sex interaction effect on parent-adolescent discordance (*F*(2, 2154) = 7.757, *p* < 0.001) were found. Figure 2 visualizes how standardized parent-adolescent discordance developed over time for boys and girls, with parents reporting slightly less symptoms than girls themselves, and slightly more symptoms than boys themselves over time.

### Longitudinal Associations between Discordance and Functional Somatic Symptoms in Early Adulthood

Linear regression analyses were conducted to test if parent-adolescent discordance at T1–T3, expressed in standardized difference scores, predicted symptom prevalence at T5. The assumption of independent residuals was met, indicating that there was no dependence between the residuals of parent-adolescent discordance across the waves. Zero order correlations between parent-reported FSS at T1–T3 and self-reported FSS at T5, and self-reported FSS at T1–T3 and self-reported FSS at T5, respectively, were in the same direction and range (see Appendix Table 5). This shows similar bivariate correlations between the reports of parent-report and self-report and the outcome.

Table 2 shows that parent-adolescent discordance at T1–T3 significantly associated with self-reported functional somatic symptoms at T5, after adjusting for sex, SES, pubertal status and gender non-contentedness (*b* = 0.234, 95% CI [0.134, 0.334], *p* < 0.001). This indicates that higher positive parent-adolescent discordance (i.e., lower parent-reported functional somatic symptoms than self-reported functional somatic symptoms) was associated with higher self-reported functional somatic symptoms at T5. The interaction between parent-adolescent discordance and sex was not significant (*b* = −0.057, 95% CI [−0.193, −0.079], *p* = 0.396). This indicates that the strength of the association between parent-adolescent discordance at T1–T3 and self-reported functional somatic symptoms at T5 does not differ between boys and girls.

**Table 2:** Linear regression analyses: longitudinal associations between parent-adolescent discordance in functional somatic symptoms at T1–T3 and self-reported functional somatic symptoms at T5

Predictor	*b (95% CI)*	*p*
Parent-adolescent discordance T1–T3	0.234 (0.134–0.334)	<0.001
Adolescent sex	−0.922 (−1.060–−0.784)	<0.001
Parent-adolescent discordance * Adolescent sex	−0.057 (−0.193–0.079)	0.396
SES	−0.311 (−0.386–−0.237)	<0.001
Gender non-contentedness	0.084 (−0.105–0.272)	0.383
Pubertal status	0.056 (0.004–0.108)	0.035

Polynomial regression analyses were conducted to test if the association between parent-reported functional somatic symptoms at T1–T3 and symptom prevalence at T5 differed by adolescents’ self-reported functional somatic symptoms at T1–T3. The assumption of independent residuals was met, indicating that there was no dependence between the residuals of parent-reported functional somatic symptoms across the waves and, likewise, the residuals of self-reported functional somatic symptoms across the waves. Model fit did not significantly improve by entering cubic main effects and quadratic interaction terms (block 3), and none of the higher-order interactions were significant. Quadratic main effects were also non-significant. This indicates that polynomial terms did not fit the data better than linear terms.

Table 3 shows that both parent-reported functional somatic symptoms at T1–T3 (*b* = 0.314, 95% CI [0.236, 0.392], *p* <0.001) and self-reported functional somatic symptoms at T1–T3 (*b* = 0.426, 95% CI [0.363, 0.489], *p* <0.001) were significant predictors of self-reported functional somatic symptoms at T5. However, the two-way interaction term between self-reported functional somatic symptoms at T1–T3 and parent-reported functional somatic symptoms at T1–T3 was not significant (*b* = −0.011, 95% CI [−0.076, 0.054], *p* = 0.729). This indicates that the strength of the association between parent-reported functional somatic symptoms at T1–T3 and self-reported functional somatic symptoms at T5 does not differ by self-reported functional somatic symptoms at T1–T3 (i.e., the association is similar for high and low self-reported functional somatic symptoms at T1–T3). The three-way interaction term between self-reported functional somatic symptoms T1–T3, parent-reported functional somatic symptoms T1–T3, and adolescent sex was also not statistically significant (*b* = 0.047, 95% CI [−0.043, 0.136], *p* = 0.306), indicating that the strength of the association between parent-reported functional somatic symptoms at T1–T3 and self-reported functional somatic symptoms at T5 does not differ by self-reported functional somatic symptoms at T1–T3 between boys and girls.

**Table 3:** Polynomial regression analyses: longitudinal associations between parent-reported and self-reported functional somatic symptoms at T1–T3 and self-reported functional somatic symptoms at T5

Predictor	*b (95% CI)*	*p*
Adolescent sex	−0.735 (−0.856–−0.614)	<0.001
SES	−0.200 (−0.273–−0.127)	<0.001
Gender non-contentedness	−0.030 (−0.210–0.150)	0.741
Pubertal status	0.052 (0.002–0.102)	0.041
Parent-reported FSS T1–T3	0.314 (0.236–0.392)	<0.001
Self-reported FSS T1–T3	0.426 (0.363–0.489)	<0.001
Parent-reported FSS T1–T3^2^	−0.006 (−0.050–0.039)	0.793
Self-reported FSS T1–T3^2^	0.034 (−0.010–0.078)	0.126
Parent-reported FSS T1–T3 * Self-reported FSS T1–T3	−0.011 (−0.076–0.054)	0.729
Parent-reported FSS T1–T3 * Self-reported FSS T1–T3 * Adolescent sex	0.047 (−0.043–0.136)	0.306

Cubic main effects and quadratic interaction terms were entered in block 3, but were dropped from the model again since model fit did not significantly improve and none of the additional interaction terms were significant. These higher-order predictors were therefore omitted from the table

*FSS* functional somatic symptoms

Post-hoc analyses revealed no significant parent-reported functional somatic symptoms at T1–T3 by sex interaction (see Appendix Table 6), indicating no difference between boys and girls in strength of the association between parent-reported functional somatic symptoms at T1–T3 and self-reported functional somatic symptoms at T5.

## Discussion

Although extensive research has shown that functional somatic symptoms are more prevalent in girls than in boys, a gap exists in the literature regarding the role of discordance in parent-reported and adolescent self-reported symptoms in the sex difference in symptom prevalence. To address this gap, this study investigated sex differences in parent-adolescent discordance in reported functional somatic symptoms and its longitudinal association with future symptom prevalence using data from four waves of a large population-based cohort.

The results showed that parent-reported and self-reported functional somatic symptoms were significantly higher in girls than in boys throughout adolescence. However, when comparing parent-report with self-report, parents reported slightly less symptoms than girls self-reported. The course of parent-adolescent discordance over adolescence differed between boys and girls, with parents increasingly reporting more symptoms in girls than girls themselves over time, and vice versa in boys. Furthermore, using standardized difference scores, it was found that lower parent-reported than self-reported symptoms contributed to symptom prevalence in early adulthood. Using polynomial regression, it was found that interactions between parent-reported and self-reported symptoms did not associate with early adulthood symptom prevalence. No sex differences were detected in the associations with early adulthood symptom prevalence.

The weak to moderate correlations between parent-reported and self-reported functional somatic symptoms that we found are consistent with previous studies assessing informant discordance, which reported correlations in the range of 0.15–0.40 (Achenbach et al., 1987; De Los Reyes et al., 2015; Hart et al., 2013; Sourander et al., 1999). An explanation for low correspondence could lie in the relatively low observability of somatic symptoms, which may even more be the case with functional somatic symptoms than in symptoms that are part of a biomedical condition. The finding that higher levels of functional somatic symptoms are reported in girls, both by themselves and by their parents, also concurs with previous work (Duhig et al., 2000; Janssens et al., 2011; van Geelen et al., 2015). The sex difference that the current study found in parent-child discordance in adolescence, however, contrasts with previous studies on parent-adolescent discordance in somatic symptoms reports, which showed no moderation effect of sex (Kröner-Herwig et al., 2009; Poulain et al., 2020). Those studies had, in contrast to the current study, cross-sectional designs and included younger participants, which may explain the different findings.

Contrary to the hypothesis, parental underestimation of symptoms was observed in girls and overestimation in boys. This finding contrasts with gender role literature on femininity, stating that girls are more susceptible to pain and more open in expressing their complaints as it is more socially accepted for women and girls to express pain (MacLean et al., 2010). An explanation might be, albeit speculative, that girls do behave increasingly according to the feminine gender role, thus more openly expressing symptoms, and boys vice versa. However, parents may not perceive this behavioral change, resulting in parental underestimation of symptoms in girls and overestimation in boys. Alternatively, transmission of symptom-related gender roles may not be captured well by studying parent-adolescent discordance, as symptom management comprises more than merely the estimation of symptom prevalence. Possibly, it would be better captured in measures reflecting social learning, such as illness behavior modeling or parental responses to child symptoms.

This is the first large epidemiological study that assessed longitudinal associations between parent-adolescent discordance and future symptom prevalence. Confirming the hypothesis, it was found that parent-adolescent discordance, expressed in standardized difference scores, contributed to future symptoms. This finding is similar to previous studies that focused on parent-child discordance in psychopathology and showed longitudinal associations with later adverse psychological outcomes, such as anxiety and depressive symptoms (Ferdinand et al., 2004; Van de Looij-Jansen et al., 2011). Notably, using polynomial regression analysis, the interaction between parent-reported and self-reported symptoms in adolescence was not significantly associated with early adulthood symptom prevalence. The results show that parent-reported symptoms in adolescence associated independently with early adulthood symptom prevalence, but the strength of this association was unaffected by self-reported symptoms in adolescence. Yet, the existence of discordance between parent-report and self-report, measured using standardized difference scores, was associated with early adulthood symptom prevalence. Contrary to the hypothesis, however, the discordance contributing to future symptoms concerned parental underestimation (parents reporting fewer symptoms than the adolescent), rather than parental overestimation (parents reporting more symptoms than the adolescent). Possibly, parents failing to identify symptoms in their children could lead to the child feeling misunderstood or overlooked, which may be harmful for their health and well-being (Van de Looij-Jansen et al., 2011). Moreover, adolescents with burdening functional somatic symptoms may not receive professional treatment in time because their parents do not see the need to take their child to a health care professional, while early interventions could prevent persistence and exacerbation of symptoms (Berezowski et al., 2022). In this way, parental underestimation of symptoms could contribute to a poor prognosis. It is worth noting that although parental underestimation of symptoms contributed to future symptom prevalence in both boys and girls, more underestimation was observed in girls. This possibly constitutes part of the explanation for the higher prevalence of functional somatic symptoms in girls.

The findings of this study, if replicated, may aid in developing preventive and treatment strategies for burdening functional somatic symptoms. Children of parents who report fewer symptoms than the children themselves, which in our study were predominantly girls, may be particularly at risk of a poor symptom prognosis. For clinicians working with adolescents with functional somatic symptoms, it is important to be aware of the limited correspondence between parent-reported and self-reported symptoms and of the risk posed by parental unawareness of symptoms. Important to mention is that high parent-reported FSS emerged as an independent risk factor for future symptom prevalence, concurring with previous studies (Janssens et al., 2014). This indicates that preventive and treatment strategies may be desirable not only in the case of parental underestimation of symptoms but also in cases of high parental perception of symptoms (regardless of the adolescent’s self-reported symptoms). These findings underline the importance of involving parents in the treatment of children and adolescents presenting with functional somatic symptoms, as has been indicated by previous research (Hulgaard et al., 2019).

### Strengths and Limitations

This study has several strengths. We used data from a large population-based cohort, which enhances the generalizability of the results (Oldehinkel et al., 2015). Moreover, we included data from four assessment waves covering the entire developmental period from adolescence into early adulthood. Another strength is the use of validated instruments to assess functional somatic symptoms by different informants, including adolescents. In addition, we included gender non-contentedness in our model. Gender, in addition to sex, may explain differences in the occurrence and trajectories of somatic symptoms (Ballering et al., 2022; Ballering et al., 2020) and may influence parent-reported and self-reported functional somatic symptoms. We have included this variable in an effort to capture an aspect of the complex interplay between sex, gender and somatic symptoms. Furthermore, we examined the longitudinal effect of informant discordance using both the approach of standardized difference scores and polynomial regression, allowing for more comprehensive, informative and nuanced results (Laird & De Los Reyes, 2013; Tackett et al., 2013).

Limitations of our study should be taken into account as well. First, parental sex could not be included in the study, as these data were unavailable. Parental sex may associate with levels of reported functional somatic symptoms in their child, as mothers and fathers may perceive symptoms in their child differently (Schroeder et al., 2010). Furthermore, functional somatic symptoms were assessed using the YSR, ASR and CBCL. Even though these questionnaires specifically assess symptoms that occur without medical cause or obvious reason, we cannot be sure that the reported somatic symptoms are not part of an explained biomedical condition. However, regarding longitudinal associations of parent-adolescent discordance and functional somatic symptoms in early adulthood, it may not matter if the symptoms are medically explained or unexplained, as similar gendered socialization processes could take place. Finally, non-random attrition was present, with more drop-outs of male and low SES participants. However, no differences in internalizing problems (including functional somatic symptoms) were found between participants who continued to participate and those who dropped out (Oldehinkel et al., 2015). Moreover, multiple imputation was used for the longitudinal analyses to handle missing data, thereby reducing the risk of bias.

### Directions for Future Research

Possibly, parental underestimation of functional somatic symptoms in their daughters partly explains the higher symptom prevalence in girls. Other factors contributing to the increasing sex difference in functional somatic symptom prevalence are thought to include differences in biological features (e.g., hormones and pain regulatory systems), symptom labeling, puberty-related increase of depressive and anxiety symptoms in girls and incidence of sexual abuse (Alloy et al., 2016; Barsky et al., 2001; Meints & Edwards, 2018; Mendle, 2014). Future research should focus on elucidating factors contributing to the growing sex difference in functional somatic symptom prevalence during adolescence. Studying sex differences in processes of social learning of illness behavior longitudinally, including assessments from an early age onwards, may further clarify the emergence of sex differences in functional somatic symptoms. Furthermore, considering the growing body of evidence showing that gender associates with health, future research should include measures of gender when studying sex differences in functional somatic symptoms in children and adolescents (Ballering et al., 2022, 2020; Pelletier et al., 2015; Schiebinger & Stefanick, 2016; Smith & Koehoorn, 2016). In addition, relations between parent-adolescent discordance and future functional somatic symptom prevalence may be different in clinical populations. Studying parent-adolescent discordance in relation to developmental trajectories of somatic symptoms in clinical samples could be informative in this respect.

## Conclusion

There is a lack of studies combining parent-reported and adolescent boys’ and girls’ self-reported functional somatic symptoms to investigate the possible influence of parent-adolescent discordance on future symptom prevalence. The current study aimed to examine sex differences in the longitudinal course of parent-adolescent discordance and its association with symptom prevalence in early adulthood. The results of this study show that parental underestimation of functional somatic symptoms contributes to increased future symptom prevalence. In our study, this association was similar for boys and girls. However, over the course of adolescence, parental underestimation was more observed in girls than in boys. This might partly explain why girls are more prone to experiencing functional somatic symptoms than boys. The findings may aid in developing preventive and treatment strategies for burdening functional somatic symptoms. Interventions may be targeted specifically at parent-child dyads that differ greatly in their reporting. Future research should seek to elucidate factors contributing to the increasing sex difference in symptom prevalence in adolescence, which are presumably biopsychosocial.

## Appendix

Tables 4–6

**Table 4:** Correlations between parent-reported and self-reported functional somatic symptoms

		Parent-reported FSS T1	Parent-reported FSS T2	Parent-reported FSS T3
Boys	Self-reported FSS T1	0.239 *p* < 0.001		
	Self-reported FSS T2		0.285 *p* < 0.001	
	Self-reported FSS T3			0.244 *p* < 0.001
Girls	Self-reported FSS T1	0.218 *p* < 0.001		
	Self-reported FSS T2		0.379 *p* < 0.001	
	Self-reported FSS T3			0.349 *p* < 0.001

*FSS* functional somatic symptoms

**Table 5:** Correlations between parent-reported functional somatic symptoms in adolescence, self-reported functional somatic symptoms in adolescence and self-reported functional somatic symptoms in early adulthood

	Self-reported FSS T5
Self-reported FSS T1–T3	0.321 *p* < 0.001
Parent-reported FSS T1–T3	0.250 *p* < 0.001

*FSS* functional somatic symptoms

**Table 6:** Post-hoc polynomial regression analyses: longitudinal associations between parent- and self-reported functional somatic symptoms at T1–T3 and self-reported functional somatic symptoms at T5

Predictor	*b (95% CI)*	*p*
Adolescent sex	−0.742 (−0.868–−0.617)	<0.001
SES	−0.201 (−0.274–−0.128)	<0.001
Gender non-contentedness	−0.031 (−0.211–0.149)	0.732
Pubertal status	0.052 (0.002–0.102)	0.040
Parent-reported FSS T1–T3	0.353 (0.230–0.76	<0.001
Self-reported FSS T1–T3	0.427 (0.364–0.490)	<0.001
Parent-reported FSS T1–T3^2^	−0.007 (−0.052–0.038)	0.753
Self-reported FSS T1–T3^2^	0.035 (−0.009–0.078)	0.121
Parent-reported FSS T1–T3 * Self-reported FSS T1–T3	−0.024 (−0.088–0.040)	0.461
Parent-reported FSS T1–T3 * Self-reported FSS T1–T3 * Adolescent sex	0.067 (−0.027–0.161)	0.162
Parent-reported FSS T1–T3 * Adolescent sex	−0.081 (−0.233–0.070)	0.273

A two-way interaction between parent-reported FSS T1–T3 and adolescent sex was now included. Cubic main effects and quadratic interaction terms were entered in block 3, but were dropped again from the model since model fit did not significantly improve and none of the additional interaction terms were significant. These higher-order predictors were therefore omitted from the table

*FSS* functional somatic symptoms

## Footnotes

1 parent-reported functional somatic symptoms; self-reported functional somatic symptoms

2 parent-reported functional somatic symptoms^2^; self-reported functional somatic symptoms^2^

3 parent-reported functional somatic symptoms at T1–T3 by self-reported functional somatic symptoms at T1–T3; parent-reported functional somatic symptoms at T1–T3 by self-reported functional somatic symptoms at T1–T3 by sex

4 parent-reported functional somatic symptoms^3^; self-reported functional somatic symptoms^3^

5 parent-reported functional somatic symptoms^2^ by self-reported functional somatic symptoms; parent-reported functional somatic symptoms by self-reported functional somatic symptoms^2^; parent-reported functional somatic symptoms^2^ by self-reported functional somatic symptoms by sex; parent-reported functional somatic symptoms by self-reported functional somatic symptoms^2^ by sex
